# Cardiovascular Events and Mortality in Patients on Hemodialysis: The Prognostic Value of the CHA_2_DS_2_-VASc Score

**DOI:** 10.3390/medicina60010144

**Published:** 2024-01-12

**Authors:** Theodoros Tourountzis, Georgios Lioulios, Fotini Stasini, Zoi Skarlatou, Stamatia Stai, Michalis Christodoulou, Eleni Moysidou, Evdoxia Ginikopoulou, Maria Stangou

**Affiliations:** 1 Protypo Dialysis Center, Hemodialysis Unit, 55535 Thessaloniki, Greece; ttourou@gmail.com (T.T.); fotini_stas@yahoo.gr (F.S.); zskarlatou@yahoo.gr (Z.S.); ginikopoulou@gmail.com (E.G.); 2 Department of Nephrology, Hippokration Hospital, School of Medicine, Aristotle University of Thessaloniki, 54642 Thessaloniki, Greece; pter43@yahoo.gr (G.L.); staimatina@yahoo.gr (S.S.); michalischristodoulou22@gmail.com (M.C.); moysidoueleni@yahoo.com (E.M.)

**Keywords:** stroke, atrial fibrillation, hemodialysis, CHA_2_DS_2_-VASc score

## Abstract

*Background and Objectives*: Cardiovascular events are the major cause of morbidity and mortality in patients on hemodialysis (HD). Identifying risk factors can help in the effort to reduce cardiovascular risk and improve life expectancy. The objective of this study was to evaluate the ability of the CHA_2_DS_2_-VASc score—the risk index of stroke in atrial fibrillation (AF)—to predict strokes, major cardiovascular events, and mortality in patients with end-stage kidney disease. *Materials and Methods*: The CHA_2_DS_2_-VASc and HAS-BLED scores (the bleeding risk from the use of anticoagulation in AF) were calculated in 237 HD patients, 99 women with a median age of 76 (15) years, at the time they commenced HD. The scores’ ability to predict long term cardiovascular morbidity and mortality was estimated, both in those with and without AF. Among the exclusion criteria were the change of dialysis method or loss of follow-up, HD due to acute renal failure, and incompliance with medical instructions, thus the sample is not representative of a broader population. *Results*: The CHA_2_DS_2_-VASc score was higher in AF (*n* = 69) compared to non-AF (*n* = 168) patients, 5 (2.5) vs. 4 (2), *p* < 0.0001, respectively. An increased CHA_2_DS_2_-VASc score was correlated with cardiovascular events, namely, heart failure (*p* = 0.007, *p* = 0.024), stroke (*p* < 0.0001, *p* < 0.0001), and risk of all-cause mortality (*p* < 0.0001, *p* < 0.0001) in AF and non-AF groups, respectively. The C statistics indicated that the referred score showed modest discrimination in AF and non-AF patients on HD for heart failure, stroke, and all-cause mortality, however for cardiovascular mortality this was found only in the AF group. *Conclusions*: An increased CHA_2_DS_2_-VASc score at the time of HD initiation can predict strokes, heart failure, and all-cause mortality in HD patients independent of the presence of AF. The risk of cardiovascular mortality could only be predicted in patients with AF.

## 1. Introduction

The need to determine cardiovascular composite end points in clinical studies led to the establishment of the term major adverse cardiovascular events (MACE). In the period 1990-2000, its use was initially restricted to describe a composite of clinical events, such as in-hospital complications following percutaneous coronary interventions. In the following years, the definition of MACE was widely applied in cardiovascular research, however with great heterogeneity, which finally revealed the practical difficulties in using a broadly comprehensive end point to describe cardiovascular complications, and forced researchers to substitute it with separate end points [1]. According to the World Health Organization, cardiovascular diseases include: coronary heart, cerebrovascular, peripheral arterial, rheumatic heart, and congenital heart disease, as well as deep vein thrombosis and pulmonary embolism [2]. Stroke is not only the second main cause of death worldwide, but it is also associated with disability. Although ischemic stroke is more frequent, hemorrhagic stroke is related to increased deaths and disability-adjusted life-years lost [3]. Atrial fibrillation (AF) is the most ordinary clinically recognized arrhythmia and is a significant cause of morbidity and mortality. Its incidence in people aged between 60–65 years is less than 1%, but that increases to 8–10% among those over 80 years of age. As the responsible cause of 15% of stroke events, it is considered to display a fivefold risk increase of stroke [4].

The CHA_2_DS_2_-VASc score has been designed and broadly applied as a useful tool that calculates the risk of stroke in patients with AF. Patients receive one or two points for each criterion, which are: congestive heart failure, arterial hypertension, age (over 75 or 65–74 years), diabetes mellitus, previous stroke/transient ischemic attack/thromboembolism, vascular disease, and gender. Patients are classified as low, intermediate, or high risk to predict the annual risk of stroke (0.2–12.2%). In the high-risk patients, the utilization of orally administrated anticoagulant medication is suggested, whereas in the moderate risk population, it can be regarded as a potential therapy [5]. Table S1 (found in the Supplementary Materials) describes the parameters that consist of and determine the CHA_2_DS_2_-VASc score. This score is not often used in patients on HD and previous studies have tried to correlate it with cardiovascular and all-cause mortality at HD [6,7,8,9,10].

Moreover, the HAS-BLED score was described to predict bleeding risk from the use of anticoagulation in patients with AF. The HAS-BLED score includes the presence of systolic blood pressure over 160 mmHg, impaired renal function, serum creatinine above 2.26 mg/dL or the need for dialysis, liver disease (cirrhosis, serum bilirubin levels two times above normal, serum transaminases levels three times above normal), history of stroke, previous bleeding/predisposition to bleeding, over 65 years of age, irregular International Normalized Ratio (INR), medication that predisposes one to bleeding, and alcohol use. It is classified into low, intermediate and high risk for bleeding, as described in Table S2 of the Supplementary Materials. Its use aim is to reduce the risk of bleeding using appropriate measures [11].

In this study we aim to evaluate the ability of the CHA_2_DS_2_-VASc score to predict not only strokes, but also, major cardiovascular events and mortality in patients with end stage kidney disease (ESKD) measured at the time started on hemodialysis (HD). In fact, the study not only tried to repurpose this risk score and expand its use as a predictive indicator of cardiovascular events in patients on HD, but also applied it to patients with AF and without AF. The present study contributes to other studies that have also focused mainly on cardiovascular and all-cause mortality, and not so often on other cardiovascular events.

## 2. Materials and Methods

### 2.1. Study Population

#### Patient Inclusion and Exclusion Criteria

The patients included in the study were adults, above 18 years old, with ESKD, on HD for at least 12 months prior to participation, in order to have a follow-up of at least one year and to avoid cases of acute renal failure temporally requiring HD [12,13,14].

The exclusion criteria were as follows: change of dialysis method (peritoneal dialysis or transplantation) during the last 12 months, incompliance with medical and dietary instructions and HD rules, patients who could not achieve adequate dialysis or were lost to follow-up.

Concerning the potential selection biases, the study had attrition and sampling ones [15]. Specifically, 73 patients were lost to follow-up or drop out (change of dialysis unit) and it is not known if they have different characteristics from those who remained in the study. Furthermore, the study did not include participants with acute renal failure (*n* = 8), other CKD stages, or with an alteration of dialysis method. Thus, for the above patients the outcome (cardiovascular event) is unknown.

### 2.2. Study Methodology and Data Extraction

The present retrospective study included patients who commenced HD during the period February 2010 until January 2021, and stayed on a regular HD method for at least 12 months. The CHA_2_DS_2_-VASc and HAS-BLED scores were calculated at the time of commencing HD. In addition, at the same time of HD initiation, demographic, clinical and laboratory data, comorbidities and information regarding medication, and HD prescription were collected from patients’ records. The duration of the follow-up period was from the time of HD initiation to either January 2021 or death.

Major cardiovascular events and deaths were recorded during their follow-up period on HD. Laboratory indices, such as serum total cholesterol, high-density lipoprotein cholesterol (HDL-C), low-density lipoprotein cholesterol (LDL-C), triglycerides and high sensitivity troponin T (TnT-hs) were also recorded at the end of the follow-up.

### 2.3. Statistical Analysis

Statistical processing and analysis were performed using the Statistical Package for Social Sciences (SPSS) 26 IBM Corp, Armonk, NY, USA, for Windows. The statistical significance threshold (p) was set at <0.05. The distribution of continuous variables was determined as normal or non, by using the Kolmogorov–Smirnov and Shapiro–Wilk tests. These variables were demonstrated as mean ± standard deviation (SD) or median (interquartile range, IQR). Means were compared with Pearson’s chi square test (x^2^), while medians with the Mann–Whitney test. The calculation of the discrimination of CHA_2_DS_2_-VASc score was made by using C statistics for each outcome. Receiver Operating Characteristics (ROC) curves were applied to estimate the incidence of the CHA_2_DS_2_-VASc score in cardiovascular diseases (CVD), cardiovascular morbidity, and mortality.

## 3. Results

### 3.1. Description of Patient Data

A total of 383 Caucasian patients were initially evaluated, however 146 of them were removed from the analysis either due to the presence of exclusion criteria or due to the lack of compliance during follow-up, resulting in 237 patients that were finally analyzed. The lifetime of the studied population was 76 (15) years old, most of them 197/237 (83.1%) being elderly, ≥65 years old. The mean value of their body mass index was 24.99 ± 5.12 kg/m^2^. Approximately half of the patients (*n* = 132/237, 55.7%) were alive at the data collection cut-off date. The median levels of serum cholesterol and triglycerides were within physiological limits. Nevertheless, the majority of the participants received statins, ezetimibe, or omega 3 fatty acids. LDL-C levels were above the recommended goals (<55 mg/dL) according to the 2019 European Society of Cardiology (ESC) and European Atherosclerosis Society (EAS) guidelines [16]. The patients’ demographics are condensed in Table 1.

### 3.2. Cardiovascular Events during Follow-Up

Acute myocardial infarction and/or peripheral arterial disease was present in almost one third of the patients, respectively. In most of the participants, the cardiovascular event was diagnosed before the initiation of HD. Approximately 1 in 4 patients (*n* = 63, 26.6%) experienced a stroke, with the majority being ischemic etiology (*n* = 60), and in 35 patients some complications or neurologic residuals were present. AF was present in 29.1% of patients, and nearly 1 in 10 developed arrhythmia within the first three months after starting dialysis. Out of the AF patients, 29 had paroxysmal and 40 had persistent, long-standing persistent or permanent AF. All patients with paroxysmal AF underwent a rhythm control strategy, i.e., pharmacological or electrical cardioversion, and none were admitted for catheter ablation. Twelve of them remained in antiarrhythmic therapy with amiodarone and seventeen received rate control therapies. Forty patients with persistent, long-standing persistent or permanent AF were also treated with rate control medication, i.e., beta-blockers, non-dihydropyridine calcium channel blockers (diltiazem, verapamil), or digoxin. None of the patients received direct oral anticoagulants (DOAC), since we started the use of them in our dialysis unit after February 2021. Almost six percent of patients developed venous thromboembolic disease, occurring before the start of HD in the vast majority of them, in the form of deep vein thrombosis. The characteristics of cardiovascular events are summarized in Table 2.

### 3.3. Correlation with Morbidity and Mortality

All patients with AF had an abnormal CHA_2_DS_2_-VASc and HAS-BLED score, as seen in Table 3. The CHA_2_DS_2_-VASc score was 5 (2.5) vs. 4 (2), *p* < 0.0001 in AF and non-AF, respectively. No significant difference between AF and non-AF patients was demonstrated regarding the HAS-BLED score.

In addition, in Table 4, the causes of death as reported in the Service for Coordination and Control of the End Stage Chronic Kidney Failure Program are listed. One third of the 105 patients who died had an underlying cardiovascular cause of death. Higher mortality was depicted in the AF group, 40/69 (58%), in comparison to the non-AF group, 65/168 (38.7%), chi-square = 7.36, *p* = 0.006.

In Table 5, the association of the CHA_2_DS_2_-VASc score with morbidity and cardiovascular and all-cause mortality in patients with and without AF (*n* = 69 and *n* = 168, respectively) is depicted. In our study population, this increased score was correlated with heart failure (*p* = 0.007, *p* = 0.024) and stroke (*p* < 0.0001, *p* < 0.0001) in AF and non-AF patients, respectively. This score may forecast the risk of all-cause mortality in patients with and without AF, *p* < 0.0001, *p* < 0.0001, respectively.

**Figure 1:**
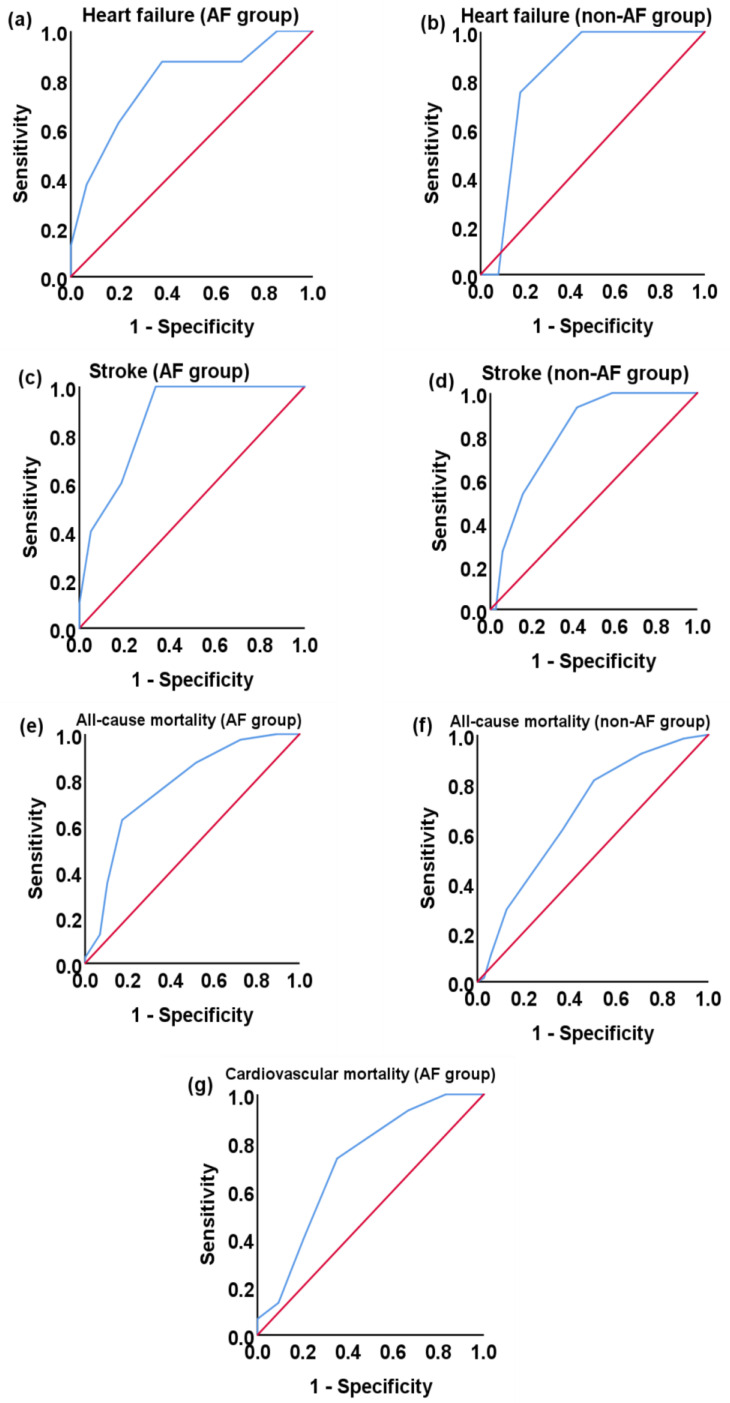
Receiver operating characteristic (ROC) curves for CHA_2_DS_2_-VASc score relationship with cardiovascular events in atrial fibrillation (AF) (**a**,**c**,**e**,**g**) and non-AF (**b**,**d**,**f**) patients on HD.

In AF and non-AF patients on HD, the C statistics indicated that the CHA_2_DS_2_-VASc score had a modest performance for cardiovascular events that were diagnosed after the commencement of HD, namely acute myocardial infarction 0.681 (95% confidence interval [CI], 0.335–1) *p* = 0.227, and 0.442 (95% CI, 0.314–0.571) *p* = 0.523, heart failure, 0.79 (95% CI, 0.613–0.967) *p* = 0.008 and 0.825 (95% CI, 0.722–0.929) *p* = 0.026, peripheral arterial disease 0.65 (95% CI, 0.499–0.801) *p* = 0.071 and 0.524 (95% CI, 0.39–0.658) *p* = 0.76, stroke, 0.864 (95% CI, 0.77–0.958) *p* < 0.0001 and 0.811 (95% CI, 0.726–0.896) *p* < 0.001, cardiovascular mortality 0.713 (95% CI, 0.581–0.845) *p* = 0.012 and 0.629 (95% CI, 0.514–0.745) *p* = 0.067, and all-cause mortality 0.771 (95% CI, 0.654–0.887) *p* < 0.0001 and 0.682 (95% CI, 0.602–0.763) *p* < 0.0001, respectively. Figure 1 shows the above relationships which are statistically significant.

Subsequently, in the present study, the incidence of stroke and AF correlated with various parameters, either demographic, clinical or laboratory, or with HD characteristics. A marginally statistically significant relationship was observed between stroke occurrence and patients with a high HAS-BLED score (*p* = 0.049). The presence of AF was related to death 2.186 (95% CI, 1.236–3.865) *p* = 0.007. An increased CHA_2_DS_2_-VASc score was associated with stroke (*p* < 0.001), cardiovascular mortality (*p* = 0.047), and a central venous catheter (*p* = 0.047). Finally, the type or treatment of AF was not significantly correlated with any cardiovascular event.

## 4. Discussion

The CHA_2_DS_2_-VASc score is a very useful tool, initially described and widely applied for the calculation of ischemic stroke risk in patients with AF, and is also used to recognize and distinguish patients who can take oral anticoagulation [5]. The score has not been widely used in patients on HD, and in validation showed only a poor performance, possibly attributed to chronic inflammation and vascular calcification commonly seen in HD patients [17]. In the present study we assessed the CHA_2_DS_2_-VASc score in patients with ESKD undergoing HD, and estimated its efficacy to predict major cardiovascular events and cardiovascular and all-cause mortality. The CHA_2_DS_2_-VASc score was calculated for the whole cohort of HD patients, while its predictive ability was separately estimated for patients with AF and without AF.

The CHA_2_DS_2_-VASc score was correlated with all-cause mortality in both groups, AF and non-AF, but was correlated with cardiovascular mortality only in patients with AF. Previous studies have shown that this score is used for the estimation of not only cardiovascular, but also of all-cause mortality in persons with chronic kidney disease (CKD) with and without AF [6,7,8]. Moreover, it was used as a predictor of three-year all-cause and cardiovascular mortality in patients on HD [9]. Furthermore, a CHA_2_DS_2_-VASc score above 3.5 and the presence of a central venous catheter at the commencement of HD may predict one-year mortality in this population [10].

In our study, the CHA_2_DS_2_-VASc score may predict the risk of stroke and heart failure. It was found that each point increment in this score was related to a higher risk of events such as myocardial infarction and stroke within the first year for patients on HD [18]. In the same population, this score was associated with stroke in patients without AF. Furthermore, a CHA_2_DS_2_-VASc score above or equal to four was related to the highest risk for cardiovascular outcomes, namely, stroke, myocardial infarction, and peripheral ischemia [19]. However, it was found that preceding ischemic strokes are non-inferior for prognosticating subsequent ones, instead of the whole CHA_2_DS_2_-VASc score in the HD population. This score is less predictive in patients without a history of cerebrovascular accidents or transient ischemic attacks [20].

In the present study, one third of the deceased patients had an underlying cardiovascular cause of death, followed by infectious diseases and cancer, with a higher mortality in the AF group. A Japanese study of patients on HD found similar results, with sudden death, pulmonary infection, and lung cancer being the causes of deaths in each category [21]. A Chinese study had almost similar results, with major reasons of death being sudden death, infection, cardiovascular, and cerebrovascular disease [22]. AF at the commencement of HD was related to an increased mortality risk of ESKD patients in all age groups [23]. AF was a risk factor for outcomes in the HD population, such as death from any cause, hospitalization, and hemorrhagic stroke [24].

Stroke was associated with decreased survival and AF. In approximately half of patients, neurological deficit was present after the event. In a meta-analysis, an inverse linear relationship was found between glomerular filtration rate and stroke. The hazard of this occurrence is enhanced by 7% for every 10 mL/min/1.73 m^2^ reduction in the glomerular filtration rate [25]. CKD patients on HD encounter a higher risk of ischemic stroke in contrast to the entire population, with AF being the main risk factor. There is also increased mortality after stroke in this population [26]. While in patients with CKD stage 2, the risk of occurrence and incidence of AF is 6%, and on HD it may exceed 25%. In patients with CKD undergoing HD, the relative risk of stroke is three times higher in the presence of AF compared to its absence [4]. A Scottish study concluded that the leading risk factors of stroke were old age, AF, history of stroke, diabetes mellitus, elevated serum phosphorus levels, low body weight, and increased systolic blood pressure [27]. Another study showed that diabetic nephropathy was a statistically significant risk factor [28]. Stroke was correlated with a reduction in functional capacity and an increment in mortality. Also, the presence of AF and an abnormal CHA_2_DS_2_-VASc score [29] were common. Moreover, it has been observed that in patients with AF, a high CHA_2_DS_2_-VASc score has been associated with an increased risk of the development of CKD and ESKD [30]. Most patients had a high HAS-BLED score, regardless of AF. However, this score showed poor predictive abilities in this population [31] and had poor discriminatory performance in patients with ESKD on HD [32].

In the present study, the CHA_2_DS_2_-VASc score correlated with the risk of cardiovascular events, such as stroke, heart failure, and cardiovascular or all-cause mortality in patients on HD. Thus, patients with an increased score may be influenced by potential strategies which try to reduce the cardiovascular risk. These interventions include regulation of common risk factors, i.e., the optimal management of diabetes mellitus, hypertension and obesity, smoking cessation, and others specific to CKD, such as anemia, malnutrition, electrolyte imbalances, and volume regulation (frequent fluid shifts in patients on HD) [33]. Other recommendations are the nutritional education of patients and health workers [34] as well as the use of lipid-lowering agents in patients on HD with hyperlipidemia [35]. A potential future investigation could be on the effect of each CHA_2_DS_2_-VASc score modifiable criteria separately (congestive heart failure, arterial hypertension, diabetes mellitus, vascular disease) on cardiovascular risk in CKD patients on HD.

Although our results are based on a retrospective analysis of patients dialyzed in a single center, we believe that the large number of patients with adequate follow-up, and the strict inclusion and exclusion criteria, may overwhelm this limitation and highlight the importance of newer applications of the CHA_2_DS_2_-VASc score. Nevertheless, the present study’s findings could benefit from a prospective validation in a cohort with more participants to reinforce the reliability of the CHA_2_DS_2_-VASc score as a predictive tool in the HD population. Furthermore, as previously mentioned, this study focused only on patients on HD, and thus the results cannot be generalized to a population with different CKD stages, kidney transplant patients, or those on peritoneal dialysis. Due to the retrospective nature of the study, correlations between the scores and the cardiovascular events were found, but causality was not established. An additional inherent limitation is the recall bias, since the collection of the data was obtained retrospectively. However, we tried to prevent this by using medical records, instead of depending on patients’ answers. Lastly, the predictive value of this specific score was not evaluated with other risk factors or potential unmeasured confounders separately, such as, diabetes mellitus, hypertension, ventricular hypertrophy, chronic volume overload, anemia, inflammation, oxidative stress, and CKD–mineral bone disorder [36], although some of them are components of the scores. The patients on HD are often not included in systematic reviews and meta-analyses, such as those that could investigate the influence of different anticoagulant therapies (warfarin, DOAC, low molecular weights heparins) in the risk reduction of stroke in patients with AF, which was also not calculated in this study.

## 5. Conclusions

In the present study, stroke coexistence with AF in HD patients highlights its potential causality. The majority of patients had increased CHA_2_DS_2_-VASc and HAS-BLED scores, making it necessary not only to receive appropriate treatment, but also to try to reduce the high bleeding risk.

The CHA_2_DS_2_-VASc score may predict the risk of cardiovascular events, such as stroke, heart failure, and all-cause mortality in AF and non-AF patients on HD. However, the likelihood of cardiovascular mortality could be prognosticated solely in patients with AF.

## Figures and Tables

**Table 1:** Patients’ demographics.

Parameter	Total	AF Group	Non-AF Group	*p*
***n***	**237**	**69**	**168**	
**Primary cause of end stage kidney disease**				
Diabetic nephropathy (%)	69 (29.1)	19 (27.5)	50 (29.8)	
Arterial hypertension (%)	22 (9.3)	9 (13)	13 (7.7)	
Glomerulonephritis or vasculitis (%)	20 (8.4)	3 (4.3)	17 (10.1)	
Polycystic kidney disease (%)	8 (3.4)	1 (1.4)	7 (4.2)	
Obstructive cause (%)	12 (5.1)	2 (2.9)	10 (6)	
Cardiorenal syndrome (%)	9 (3.8)	8 (11.6)	1 (0.6)	
Cancer (%)	4 (1.7)	1 (1.4)	3 (1.8)	
Unknown cause (%)	93 (39.2)	26 (37.7)	67 (39.9)	
**At HD initiation**				
Age (IQR), years	73 (16)	76 (11.5)	72 (18.5)	0.384
Gender (%), female/male	99 (41.8)/138 (58.2)	25 (36.2)/44 (63.8)	74 (44)/94 (56)	0.268
**At time of evaluation**				
Time since staring on HD (IQR), months	36 (44)	34 (37)	37 (46.75)	0.032
Age (IQR), years	76 (15)	79 (12)	75 (17.75)	0.067
BMI (±SD), kg/m^2^	24.99 (±5.12)	25.31 (±5.34)	24.86 (±5.04)	0.568
BMI classification (%), underweight/physiological/overweight/type I obesity/type II obesity/type III obesity	41 (17.3)/90 (38)/66 (27.8)/32(13.5)/7 (3)/1 (0.4)	13 (18.8)/22 (31.9)/20 (29)/11 (15.9)/3 (4.3)/0 (0)	28 (16.7)/68 (40.5)/46 (27.4)/21 (12.5)/4 (2.4)/1 (0.6)	
Survival (%), alive/dead	132 (55.7)/105 (44.3)	29 (42)/40 (58)	103 (61.3)/65 (38.7)	0.007
**Comorbidities**				
Diabetes mellitus (%)	121 (51.1)	43 (62.3)	78 (46.4)	0.026
Arterial hypertension (%)	212 (89.5)	63 (91.3)	149 (88.7)	0.552
Dyslipidemia (%)	161 (67.9)	47 (68.1)	114 (67.9)	0.969
Cancer (%)	66 (27.8)	22 (31.9)	44 (26.2)	0.374
Chronic obstructive pulmonary disease (%)	35 (14.8)	11 (15.9)	24 (14.3)	0.744
Hypothyroidism (%)	43 (18.1)	7 (10.1)	36 (21.4)	0.041
Secondary hyperparathyroidism (%)	59 (24.9)	16 (23.2)	43 (25.6)	0.697
Cholesterol (IQR), mg/dL	152 (54.5)	147 (63.5)	152 (49.5)	0.122
HDL-C (IQR), mg/dL	42 (18)	39 (18)	43.5 (17)	0.113
HDL-C (%), <40/≥40	105 (44,3)/132 (55,7)	40 (58)/29 (42)	65 (38.7)/103 (61.3)	0.007
LDL-C (IQR), mg/dL	80 (47)	80 (54.1)	80.1 (43.75)	0.346
Triglycerides (IQR), mg/dL	140 (85.5)	142 (91.5)	139.5 (80)	0.569
TnT-hs (IQR), ng/L	63 (64.85)	79.9 (63.25)	59.05 (62.03)	0.004

Atrial fibrillation (AF); standard deviation (SD); interquartile range (IQR); hemodialysis (HD); body mass index (BMI); high density lipoprotein cholesterol (HDL-C); low density lipoprotein cholesterol (LDL-C); troponin T high sensitivity (TnT-hs).

**Table 2:** Characteristics of cardiovascular events in patients.

Cardiovascular Event	Total (*n*)	AF Group (*n*)	Non-AF Group (*n*)	*p*
Acute myocardial infarction	82	35	47	0.001
Diagnosis of acute myocardial infarction after initiation of HD	15	4	11	0.829
Heart failure	50	30	20	<0.001
Diagnosis of heart failure after initiation of HD	12	8	4	0.003
Peripheral arterial disease	80	32	48	0.008
Diagnosis of peripheral arterial disease after initiation of HD	31	16	15	0.003
Stroke (ischemic/hemorrhagic)	60/3	22/3	38/0	0.031
Complication or neurological residual from stroke	35	15	20	0.053
Diagnosis of stroke after initiation of HD	25	10	15	0.207
Venous thromboembolic disease	14	4	10	0.963
Pacemaker or defibrillator	13	8	5	0.008
Prosthetic valve	4	3	1	0.042

Atrial fibrillation (AF); hemodialysis (HD).

**Table 3:** CHA_2_DS_2_-VASc and HAS-BLED score of the population study.

Parameter	Total	AF Group	Non-AF Group	*p*
CHA_2_DS_2_-VASc score (IQR)	5 (3)	5 (2.5)	4 (2)	<0.0001
Classification of CHA_2_DS_2_-VASc score (*n*), low/intermediate/high	2/11/224	0/1/68	2/10/156	0.209
HAS-BLED score (IQR)	4 (1)	4 (1)	4 (1)	0.204
Classification of HAS-BLED score (*n*), low/intermediate/high	0/16/221	0/2/67	0/14/154	0.13

Atrial fibrillation (AF); interquartile range (IQR).

**Table 4:** Causes of death of patients in the study.

Parameter	Total (*n*)	AF Group (*n*)	Non-AF Group (*n*)
Cancer	19	4	15
Infection	4	1	3
Septicemia	18	6	12
Surgical	5	3	2
Cachexia	1	0	1
Dementia	1	1	0
Chronic obstructive pulmonary disease	1	1	0
Other	11	5	6
Unknown	11	4	7
Cardiovascular (cardiac arrest/AMI/ stroke/HF/APE/ruptured aneurysm/PE)	(13/6/11/1/1/1/1)	(6/2/6/1/0/0/0)	(7/4/5/0/1/1/1)

Atrial fibrillation (AF); acute myocardial infarction (AMI); heart failure (HF); acute pulmonary edema (APE); pulmonary embolism (PE).

**Table 5:** Correlation of CHA_2_DS_2_-VASc score with cardiovascular events and mortality in patients with and without AF after the starting of hemodialysis *.

	AF Group			Non-AF Group		
Event	Yes	No	*p*	Yes	No	*p*
Acute myocardial infarction	7 (4–7.75)	5 (4–6)	0.218	4 (3–5)	4 (3–5)	0.516
Heart failure	7 (6–8)	5 (4–6)	0.007	6 (5.25–6)	4 (3–5)	0.024
Peripheral arterial disease	6 (5–7)	5 (4–6)	0.066	4 (3–5)	4 (3–5)	0.756
Stroke	7 (6–8)	5 (4–6)	<0.0001	6 (5–7)	4 (3–5)	<0.0001
Cardiovascular mortality	6 (5–7)	6 (5–7)	0.33	5 (4–5)	5 (4–6)	0.988
All-cause mortality	6 (5–7)	5 (3–5)	<0.0001	5 (4–6)	4 (2–5)	<0.0001

* The numbers are the median value of CHA_2_DS_2_-VASc score and in parentheses is demonstrated the lower and upper IQR. Atrial fibrillation (AF); interquartile range (IQR).

## Data Availability

The data presented and analyzed during the current study are available from the corresponding author on reasonable request.

## Supplementary Materials

The following supporting information can be downloaded at: https://www.mdpi.com/article/10.3390/medicina60010144/s1, Table S1: CHA_2_DS_2_-VASc score, Table S2: HAS-BLED score. References [37,38] are cited in Supplementary Materials.
