# Supplementary material for: Cardiovascular Events and Mortality in Patients on Hemodialysis: The Prognostic Value of the CHA2DS2-VASc Score
Source: Medicina (Kaunas). 2024 Jan 12;60(1):144. doi: 10.3390/medicina60010144 (PMC10820266; doi:10.3390/medicina60010144)
Supplement: Supplementary file 1 [file medicina-60-00144-s001.zip › medicina-2792360-supplementary.pdf]

Supplemental Table S1. CHA_2DS_2-VASc score [1]

Risk factor	Score
Congestive heart failure / left ventricular dysfunction	1
Hypertension	1
Age ≥ 75 years or older	2
Diabetes mellitus	1
Stroke / transient ischemic attack / thromboembolism	2
Vascular disease (prior myocardial infarction, peripheral artery disease, aortic plaque)	1
Age 65-74 years	1
Sex category (i.e., female gender)	1
Maximum score	9

Supplemental Table S2. HAS-BLED score [2]

Risk factor	Score
Hypertension (uncontrolled, >160 mmHg systolic)	1
Abnormal renal (dialysis, renal transplant, serum creatinine >2.26 mg/dL or >200 µmol/L) and liver function (cirrhosis or bilirubin >2x normal or transaminases/ alkaline phosphatase >3x normal) (1 point each)	1 or 2
Stroke (prior history of stroke)	1
Bleeding (prior major bleeding or predisposition to bleeding)	1
Labile INR (unstable/high INR), time in therapeutic range < 60%)	1
Elderly (age > 65 years)	1
Drugs or alcohol [medication usage predisposing to bleeding: (antiplatelet agents, non-steroidal anti-inflammatory drugs) or prior alcohol or drug usage history (≥ 8 drinks/week)] (1 point each)	1 or 2
Maximum score	9

1. Lip, G.Y.H.; Nieuwlaat, R.; Pisters, R.; Lane, D.A.; Crijns, H.J.G.M. Refining Clinical Risk Stratification for Predicting Stroke and Thromboembolism in Atrial Fibrillation Using a Novel Risk Factor-Based Approach: The Euro Heart Survey on Atrial Fibrillation. *Chest* **2010**, *137*, 263–272, doi:10.1378/chest.09-1584.
2. Pisters, R.; Lane, D.A.; Nieuwlaat, R.; de Vos, C.B.; Crijns, H.J.G.M.; Lip, G.Y.H. A Novel User-Friendly Score (HAS-BLED) to Assess 1-Year Risk of Major Bleeding in Patients with Atrial Fibrillation: The Euro Heart Survey. *Chest* **2010**, *138*, 1093–1100, doi:10.1378/chest.10-0134.
